# Myopia and Metabolomics: A Comparative Study of Aqueous Humor and Serum Metabolites in Myopic Adults Undergoing Cataract Surgery

**DOI:** 10.3390/ijms26178557

**Published:** 2025-09-03

**Authors:** Emil Tomasz Grochowski, Adrian Godlewski, Karolina Pietrowska, Wioleta Gosk, Malgorzata Wojnar, Joanna Konopinska, Adam Kretowski, Michal Ciborowski, Diana Anna Dmuchowska

**Affiliations:** 1 Department of Ophthalmology, Medical University of Bialystok, M. Sklodowskiej Curie 24a, 15-276 Bialystok, Poland; 2 Metabolomics and Proteomics Laboratory, Clinical Research Center, Medical University of Bialystok, M. Sklodowskiej Curie 24a, 15-276 Bialystok, Polandmichal.ciborowski@umb.edu.pl (M.C.); 3 Department of Endocrinology, Diabetology and Internal Medicine, Medical University of Bialystok, M. Sklodowskiej Curie 24a, 15-276 Bialystok, Poland

**Keywords:** myopia, ophthalmology, metabolomics, aqueous humor (AH), serum, symmetric dimethylarginine (SDMA), taurine, cataract

## Abstract

This study aims to evaluate aqueous humor (AH) and serum metabolite concentrations in myopic and control adults undergoing cataract surgery and to correlate them with axial length (AL) to gain a better insight into the pathogenesis at both the local and systemic levels. Ninety-three patients were assigned to the myopic (n = 52) and control (n = 41) groups. Venous blood and aqueous humor samples were collected and analyzed by means of targeted metabolomics based on liquid chromatography-tandem mass spectrometry. The univariate analysis has revealed distinct metabolomic profiles between the myopic and control groups in AH but not in serum. In the AH of myopic patients, symmetric dimethylarginine (SDMA) and taurine concentrations were significantly lower. SDMA and taurine levels in the AH correlated negatively with the AL. These findings account for the insight into the local metabolic alterations in the case of myopia, potentially leading to novel therapeutic targets.

## 1. Introduction

Myopia is a widespread refractive error affecting millions of people worldwide and is a leading cause of blindness. According to projections by Holden et al. [1], it is anticipated that by the year 2050, half of the global population will be affected by myopia, while 10% will experience high myopia. This may cause a drastic increase in vision impairment due to retinal detachment, myopic macular degeneration, glaucoma, and cataracts, with the risk of those conditions increasing as the grade of myopia worsens. It is projected that the number of individuals experiencing vision loss due to high myopia will increase sevenfold from the year 2000 to 2050, positioning myopia as a leading cause of permanent blindness globally. Furthermore, those projections may be optimistic, as a drastic decrease in time spent outdoors due to the COVID-19 pandemic-related home confinement has been associated with additional myopic shifts in several reports [2,3,4,5], which will probably further impact the global prevalence of myopia. Specifically, the reduction in time spent outdoors and an increase in time spent performing near work (reading, using mobile devices) have been related to the growth of the occurrence of myopia.

Emmetropization is a complex process of obtaining the optimal size and refractive power by the eye [6]. Several animal models have been developed through the decades in order to study potential mechanisms disrupting that process and thus causing refractive errors. In short, various visual stimuli trigger signaling substances from the retina, through the choroid, to the sclera, resulting in appropriate remodeling during eye growth. The axial length (AL) is the distance from the corneal surface to an interference peak corresponding to the retinal pigment epithelium/Bruch’s membrane, and this is expressed in millimeters. Disruptions in emmetropization might cause a disproportionate increase in the AL of the eye, resulting in myopia.

Due to the alterations in the refractive power of the lens during cataract development, AL is favored for assessing myopia in senile patients. Because age-related lens opacification can alter refraction independently of eye size—nuclear sclerosis typically causing a myopic shift, and cortical opacities sometimes inducing hyperopic or astigmatic changes—we used AL as the primary indicator of myopia in older participants. AL captures the structural component of myopia and is less susceptible to cataract-related bias than spherical equivalent. In the population-based prospective cohort study, the mean AL for emmetropia was 23.30 mm (SD = 0.85; 90% range: 21.95 to 24.71), while for myopia (defined as the spherical equivalent of ≤−0.5 D) it was 24.62 mm (SD = 1.19; 90% range: 22.86 to 26.58 mm [7,8]). AL > 26 mm is commonly accepted as an indicator of high myopia [9].

So far, a definitive cure or cause-and-effect treatment strategy has not yet been established. Possible treatment options for slowing down the development of myopia include topical atropine eye drops in various concentrations (0.01–1%) and various optical treatments [10,11,12]. However, those interventions are prone to rebound effects after cessation of intervention [13]. They are based on limited data on long-term effectiveness that have mostly been gathered in Asian populations [14]. Understanding the metabolic changes that occur in patients with myopia could provide insight into the pathogenesis of myopia and potentially lead to new treatment options.

Applying metabolomics could potentially facilitate the discovery of new biomarkers and metabolic pathways and prompt the development of predictive, preventive, personalized medicine [15]. Serum from myopic patients, due to ease of collection, has been studied more extensively [16,17,18,19,20]. Only a few of the previous investigations have uncovered changes in the metabolomic profiles of myopic individuals based on the aqueous humor [21,22,23], vitreous humor [24], or cornea [25]. In the metabolomic studies conducted so far on myopia, various analytical platforms have been applied, including gas chromatography-mass spectrometry (GC-MS) [18,19,22], liquid chromatography-mass spectrometry (LC-MS) [16,17,20,21,23,25], capillary electrophoresis-mass spectrometry (CE-MS) [21], as well as divergent approaches to data analyses. Therefore, a variety of compounds and pathways have been found to be affected due to the diverse affinity of each analytical approach. Only among the amino acids, many are potentially altered in myopia: taurine, alanine, arginine, glutamate, aspartate, proline, tryptophan, histidine, valine, leucine, isoleucine, tyrosine, glutamine, lysine, serine, methionine, threonine, cysteine, glycine, and phenylalanine [26]. Together with other metabolites, the full list would contain hundreds of potentially involved metabolites.

The comprehensive metabolic pathway analysis that we performed in our review of the myopia studies has revealed that sphingolipid metabolism is the most significantly impacted metabolic pathway in human serum, while arginine biosynthesis is the most significantly impacted metabolic pathway in AH [27]. As there is no consensus on whether the pathogenesis of myopia occurs only at the local or also at the systemic level [6], there is a need for research on the comparison between the metabolite levels in the AH and serum of the same myopic patients. Although there are studies investigating metabolomic profiles of various human samples in the case of myopia, there are none yet to analyze a variety of tissues from the same patient using the same methodology, which may be crucial for a better understanding of the disease pathogenesis. We compared the metabolite concentrations in the AH and serum acquired from the myopic patients and control adults, both groups undergoing cataract surgery. Additionally, we correlated metabolomics data with the AL to gain a deeper understanding of the etiology of myopia at both the local and systemic levels.

## 2. Results

### 2.1. Patients’ Characteristics

Relevant patient information is presented in Table 1. For further details regarding the patients’ comorbidities and medication used, please see Supplementary File S1.

### 2.2. Aqueous Humor

**Figure 1:**
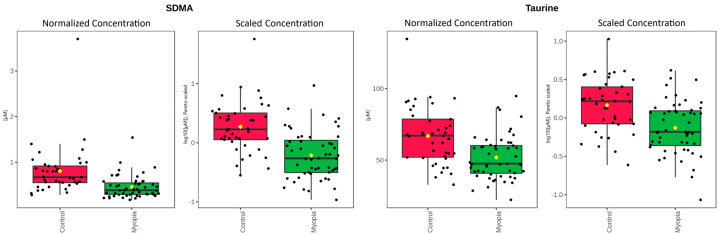
SDMA and taurine concentrations in AH. Scaled concentrations are after log data transformation and Pareto data scaling.

Out of 40 metabolites that passed filtering, two—symmetric dimethylarginine (SDMA) and taurine (Figure 1)—were found to be statistically significant (Table 2). For a full list of metabolites used for the purpose of the statistical analysis, please see Supplementary File S2.

**Figure 2:**
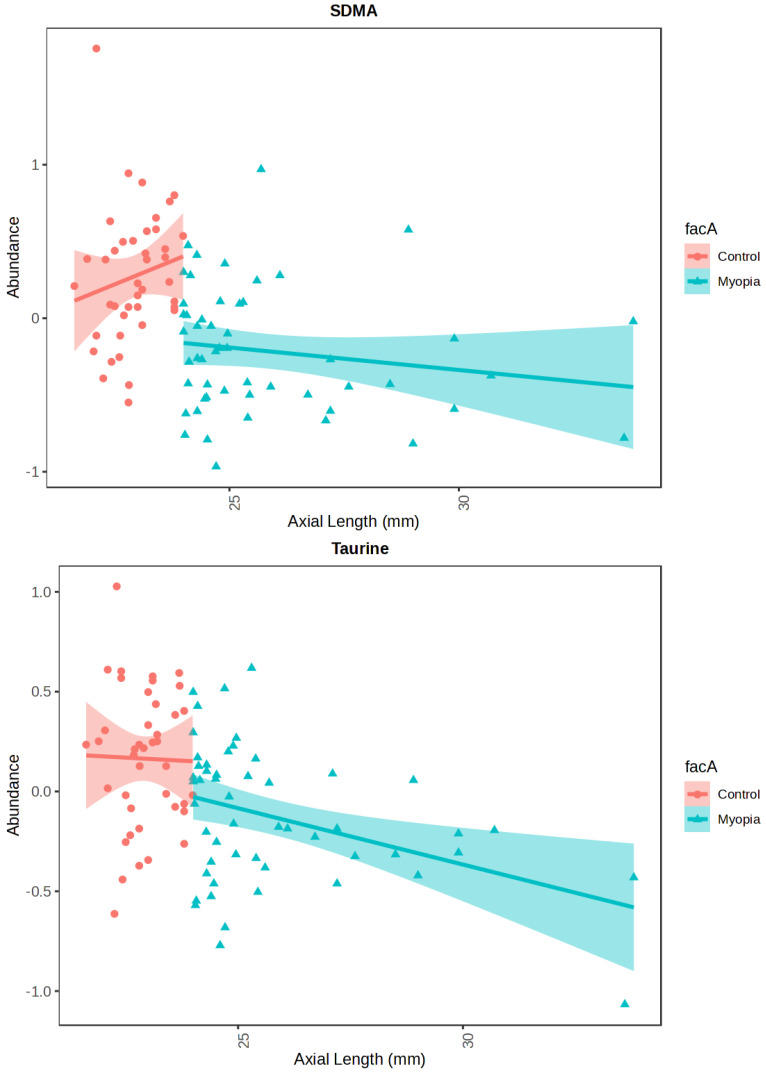
Linear model with covariate adjustment for SDMA and taurine, AL as a secondary factor. Red circles represent control samples, while blue triangles myopia samples.

**Figure 3:**
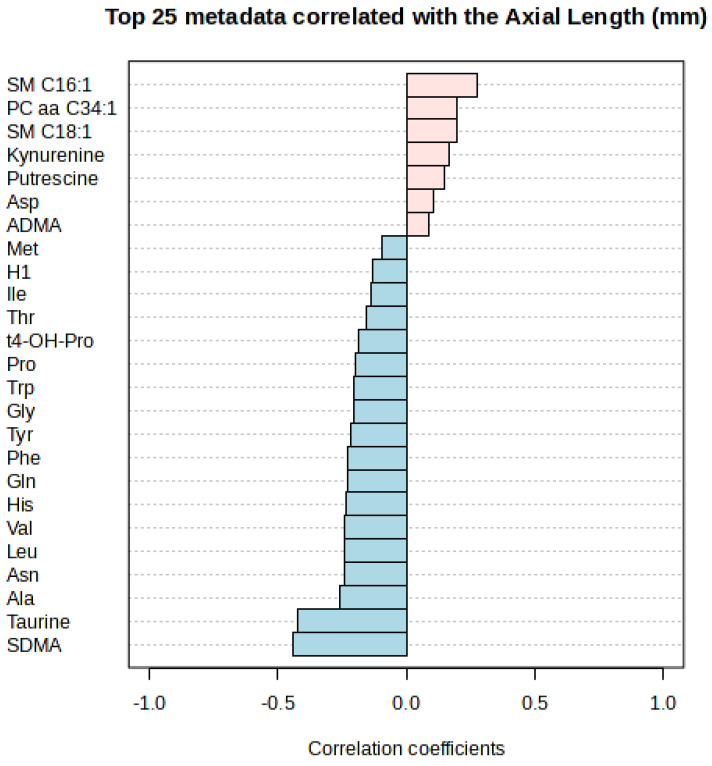
Correlation of AH metabolites with the AL. Pink represents positive correlation, while blue negative correlation.

Additionally, we utilized linear models with covariate adjustment to evaluate the impact of the AL on the abundance of the investigated metabolites in the AH independently in either group (Figure 2, Table 3). The metabolites detected in the AH were correlated with the AL by means of Spearman’s rank correlation (refer to Figure 3). Only SDMA and taurine met the FDR threshold of less than 0.05 (please see Table 4).

### 2.3. Serum

Out of 133 metabolites that passed filtering of the serum data, none was found significant after the statistical analysis (*p*-value < 0.05). For a full list, please see Supplementary File S3.

As the AH analysis has revealed two significant metabolites, we have decided to carefully check their measurements in the serum samples. The SDMA serum concentrations did not pass the initial data filtering (signals < LOD in 22.6% of the samples). The analysis of the available data has shown no differences in the SDMA serum concentration between the studied groups (*p* = 0.81). The taurine serum concentrations did not correlate with the AH levels in either cohort (Table 5).

## 3. Discussion

We have aimed to evaluate the metabolome of the serum and AH acquired from the myopic and control patients. As anticipated, metabolites were detected more abundantly in the serum than in the AH. We had previously observed similar proportions of the detected metabolites by means of the same methodology in a different study devoted to diabetes [28]. The present study has enabled us to identify the differentiating metabolites in the AH but not in the serum. Given that emmetropization is a process regulated at the local level in the eye, this outcome has somewhat been expected.

There is considerable evidence that substances flow from the posterior to the anterior part of the eye. Therefore, the metabolic composition of the AH may reflect anterior segment ocular diseases as well as posterior segment eye diseases, such as retinal disorders. It has been shown for various diseases that the results obtained for the same molecules measured in the aqueous and vitreous humor are correlated [29,30,31,32,33,34].

In the aqueous humor (AH) of myopic patients, we observed a significant reduction in SDMA concentration compared to control patients (Figure 1, Table 2). This compound also showed a negative correlation with AL (Figure 3, Table 4). Surprisingly, while SDMA concentration decreased in the myopic group along with AL, an increase in SDMA concentration was observed in the control group (Figure 2, Table 3). SDMA and asymmetric dimethylarginine (ADMA) are non-coded, toxic amino acids generated through the post-translational modification of arginine. These substances function as uremic toxins and impede nitric oxide production. They are involved in several diseases associated with dysfunction of the endothelium, such as coronary artery disease, stroke, hypertension, polycystic ovary syndrome, hyperuricemia, diabetes, and preeclampsia [35]. SDMA, possessing a methyl group on each of its terminal guanidine nitrogens, is structurally analogous to ADMA.

Recent research has linked arginine methylation to neurodegenerative diseases, neurodevelopmental disorders, depression, and schizophrenia [36]. SDMA is a significant indicator of renal health in both animals and humans. ADMA and SDMA independently indicate risk for mortality and cardiovascular disease [37]. Changes in the ADMA and SDMA concentrations in plasma most often are not reflected in intracellular levels in various tissues [35]. It is essential to concurrently assess systemic and tissue levels of ADMA and SDMA in order to clarify the relative significance of the mechanisms governing homeostasis.

Although there is no reported association between SDMA and myopia in scientific literature, it is worth noting that their precursor arginine (and citrulline, which is related to arginine through nitric oxide synthase) has been found to be associated with myopia, as they were substantially more abundant in the AH from the high myopia patients [21]. Arginine biosynthesis is the most significantly impacted metabolic pathway in AH in our review of omics research in myopia [27].

Some limited data on SDMA emerge in other studies concerning a different eye disease—glaucoma. The SDMA concentrations were elevated in the AH but not in the plasma of the primary open-angle glaucoma patients [38]. The same study did not find such a connection between ADMA and L-arginine concentrations in the AH and plasma. In contrast, another study with a more robust cohort (though restricted to the ADMA serum measurements) reported significantly higher ADMA concentrations in advanced glaucoma [39].

Our findings also reveal a substantial decrease in the level of taurine in the AH of individuals with myopia (Figure 1, Table 2) and its negative correlation with the AL (Figure 3, Table 4). The taurine concentration decreases with the AL in both cohorts (Figure 2, Table 3). Taurine is a non-essential amino acid for mammals and conditionally essential for infants, especially premature ones [40,41,42]; however, its main source remains dietary uptake. Taurine is non-proteinogenic, and as such it is not combined into proteins. Its impact on various aspects of health and disease has been extensively studied throughout the last decades; however, a complete summary of those findings is beyond the scope of this study. Most studies attribute its main function to protection against oxidative stress, as it is a potent and abundant antioxidant [43]. Oxidative stress has been associated with the development of myopia and its complications [44]. This might explain other comorbidities associated with myopia, such as a higher prevalence of glaucoma and earlier cataract development.

Taurine, among other aspects, is heavily associated with retinal function and development. Taurine is present in all ocular tissues. Rat eye tissue extracts reveal that taurine is the most prevalent amino acid in the retina, vitreous, lens, cornea, iris, and ciliary body [45]. High intercellular levels of taurine are possible due to the function of the sodium-dependent taurine transporter (TauT) in the cell membrane. TauT activity and expression in retinal pigment epithelium (RPE), ganglion, and Müller cells are regulated by hyperosmolarity [46]. Experimentally induced taurine deficiency results, among other pathologies [47], in retinal degeneration in *(taut-/-)* mice [48]. Cats are incapable of taurine biosynthesis (contrary to mice and most adult mammals), and when fed a taurine-free diet, they develop retinal degeneration, regardless of light deprivation [49]. Taurine and hypotaurine metabolism was found to be significantly down-regulated in the sclera of lens-induced myopic rabbits [50]. The same study reported that taurine concentrations were highest in the retina, followed by the choroid, and then the sclera.

A decrease in taurine levels in humans is associated with various age-linked illnesses, like obesity, diabetes, and inflammation [51]. SLC6A6 mutations in the taurine biosynthesis gene cause cardiomyopathy, and more specifically to the eye, retinal degeneration [52]. In one of our recent studies, we reported that taurine was the only metabolite significantly varying in concentration between fellow eyes [53]. Taurine and hypotaurine metabolic pathways changed in the AH in different grades of axial myopia [23].

It may be worth highlighting that sphingomyelin SM C16:1 shows a positive correlation with AL, as shown in Figure 3. Although this metabolite may not have reached statistical significance, its apparent association with AL suggests it could be of interest for further investigation, as sphingolipid metabolism is the most significantly impacted metabolic pathway in human serum of myopia studies [27].

One limitation of our study is that the participants were exclusively of Polish nationality, which may affect the direct applicability of the results to other populations, such as those in Asia, where myopia is most prevalent and extensively studied. Another limitation is the measurement of only specific metabolites available in the AbsoluteIDQ^®^ p180 kit. An untargeted metabolomics study could reveal significant differences in metabolites not covered by the kit. Still, utilizing such a kit allows for an absolute quantification, which is needed for more practical interpretation of metabolomics results [54]. Therefore, applying a quantitative approach, although for a limited number of metabolites, may be considered a strength of our research. Moreover, data sets like those in our study, which have more balanced case-to-variable ratios, are better for data modeling and less prone to overfitting [55]. The other strength is a relatively numerous study cohort, also homogenous in terms of the presence of cataracts, as none of the included patients underwent refractive lens exchange.

## 4. Materials and Methods

### 4.1. Sample Collection and Study Participants

Between 21 January and 2 December 2021, serum and AH samples were collected from 93 patients undergoing cataract surgery at the Ophthalmology Department of the Medical University of Bialystok in Poland. In accordance with current consensus (e.g., World Health Organization), we define myopia by spherical equivalent (SE) ≤ −0.50 D. For the specific aims of our targeted metabolomics study in cataract surgery patients, we phenotype axial myopia using AL as a cataract-independent structural marker to ensure stable patient stratification. This approach aligns with clinical management practice that monitors axial elongation alongside SE. Samples were divided into two groups according to the AL of the operated eye: myopia ≥ 24 mm (n = 52) and control < 24 mm (n = 41). The information on comorbidities and medications of the participants was obtained from written certificates provided by leading physicians or, when such documents were unavailable, through self-reports. The exclusion criteria included the presence of other eye diseases (excluding cataract and myopia) and a history of ophthalmic surgery or trauma.

Sample collection was performed essentially as described in detail previously [28,53,56]. In short, 2.7 mL of venous blood was collected upon admission, centrifuged, and the separated serum was frozen at −80 °C for metabolomic analysis. Twenty minutes before surgery, phenylephrine, tropicamide, diclofenac, levofloxacin, timolol, and proxymetacaine hydrochloride were administered topically. Mild sedation was acquired by oral hydroxyzine in a 25 mg dose 60 min before surgery. Two minutes before the procedure, eyes were disinfected with 5% ophthalmic povidone-iodine. A 30 G needle was used to puncture the anterior chamber and aspirate approximately 100 µL of AH, which was then frozen at −80 °C until analysis. Surgeries occurred between 8 am and 12 pm.

### 4.2. Metabolomic Analysis

A targeted metabolomic assessment of serum and AH samples was conducted using the AbsoluteIDQ^®^ p180 kit (Biocrates, Life Sciences AG, Innsbruck, Austria). This kit quantitatively measures 188 metabolites, including biogenic amines, acylcarnitines, amino acids, phosphatidylcholines, lysophosphatidylcholines, hexoses, and sphingolipids. The sampling procedure was completed in accordance with the producer’s user manual, with minor modifications already published [28]. As recommended by the manufacturer, a standard volume of 10 µL was used for serum. In the case of AH, 30 µL of the sample was used, following the optimization performed by our group [56]. The details of the sample treatment and analyses can be found in our previous publications [28,53,56]. In short, on the day of analysis, samples were thawed on ice. Quality control (QC) samples, internal standard mixture (ISTD), and calibration standards were dissolved in water and then shaken together with the biological material for 15 min. Ten µL of ISTD was added to each well of a 96-well reaction plate. QC samples, blank samples, and calibration standards were added according to the scheme. The reaction plate was evaporated in a vacuum concentrator and then derivatized. Metabolite derivatization involved the addition of 50 µL of 5% phenyl isothiocyanate prepared in a mixture of ethanol, pyridine, and water (1:1:1 *v*/*v*/*v*), and incubation for 30 min at room temperature. After evaporation of the reaction mixture, analytes were extracted from the filters using 5 mmol/L ammonium acetate in methanol. After centrifugation, the extract was split into two parts: for chromatographic analysis, 150 µL of water was added to 150 µL of extract; for flow injection analysis, the extract was diluted with methanol plus a mobile phase modifier at a ratio of 1:49. Metabolites were measured using an ultra-high performance liquid chromatograph (1290 Infinity II, Agilent Technologies, Santa Clara, CA, USA) coupled with a tandem mass spectrometer (6470 LC-MS/MS, Agilent Technologies, Santa Clara, CA, USA) operating in multiple reaction monitoring mode in positive polarity (ESI+).

Raw spectral data processing and concentration estimation were performed with MetIDQ software (Oxygen DB110-3005, Biocrates, Life Science AG, Innsbruck, Austria). The data generated was normalized in accordance with the producer’s recommendations, considering the median target value in quality control samples at level 2. Intra-plate data normalization was applied based on the target values of the quality control (QC) sample level 2 (concentration equal to the geometric center of the quantitative curve range for a given metabolite). For this purpose, correction factors were calculated by dividing the median QC2 metabolite concentration by the target concentration (specified by the manufacturer) for each kit. Metabolite concentrations were normalized by dividing each value by the calculated correction factor for each metabolite from all kits. Inter-plate normalization was performed by calculating the correction factor for each kit. For this purpose, the median metabolite concentrations for a single set were divided by the median metabolite concentrations from all sets, separately for each metabolite. Finally, metabolite concentrations were normalized by dividing each concentration value by the calculated correction factor. The data matrix was filtered to include only metabolites appearing in over 80% of instances. Following normalization of the data using quality control samples, we excluded metabolites with a coefficient of variation above 30%. Afterward, we substituted any remaining absent measurements with values determined from calibration curves, considering both below the lowest and above the highest points on these curves. The AH measurements were divided by three to account for the increased volume of the sample utilized in comparison to serum. The data matrix, containing metabolite concentrations for serum and AH samples, respectively, was submitted for statistical testing.

### 4.3. Statistical Analysis

Depending on the distribution of the data (checked by the Shapiro–Wilk test), a *t*-test was used for calculating the age and body mass index (BMI), whereas the Mann–Whitney U test was used for the AL. The chi-squared test was used for analyzing the sex distribution; *p* < 0.05 was considered statistically significant. Statistical analyses of metabolomics data were performed by means of Metaboanalyst 6.0 (https://www.metaboanalyst.ca accessed on 3 August 2024) with settings: no sample normalization, log data transformation, and Pareto data scaling. The obtained *p*-values were corrected by means of the Benjamini–Hochberg false discovery rate (FDR). Spearman’s rank correlation was used for determining the association of each metabolite with the axial length of the eye. The level of statistical significance was set at 95% (*p* < 0.05). In addition, linear models with covariate adjustment (Limma) [57] were developed with the AL serving as a secondary factor. Both supervised and unsupervised multivariate analyses were also performed. However, the parameters of the models obtained were inadequate.

## 5. Conclusions

Our goal has been to assess the metabolome of the serum and AH of the myopic patients. Having applied a targeted methodology, we have displayed the SDMA and taurine concentrations to be lower in the myopic AH, but we have found no such differences in serum. Consequently, as far as the aforementioned metabolites are concerned, their changes point to the pathophysiology at the local and not the systemic level. We have also exhibited the negative linear correlation between the AL and the SDMA and taurine concentrations. Additional investigation is necessary to clarify the place of these metabolites in the development of myopia. Presumably, a disruption of the mechanisms controlling oxidative stress in the eyes of the myopic patients is involved. Further experimental validation would be needed to confirm this assumption.

## Figures and Tables

**Table 1:** Baseline patients’ characteristics.

Characteristic	Myopia	Control	*p*-Value
Number of patients	52	41	
Sex, female, *n* (%)	28 (54)	27 (66)	0.16
Age, mean ± SD (years)	68.14 ± 10.36	70.46 ± 8.52	0.25
BMI, mean ± SD	29.81 ± 5.82	28.08 ± 4.57	0.12
AL, median [Q1;Q3] (mm)	24.85 [24.3; 26.25]	22.9 [22.5; 23.4]	<0.0001

BMI, body mass index; AL, axial length of the eye.

**Table 2:** Mean concentrations of significant AH metabolites.

	Myopia (µM) [SD]	Control (µM) [SD]	*p*-Value	FDR
SDMA	0.462 [0.25]	0.810 [0.55]	<0.0001	<0.0001
Taurine	51.865 [15.4]	66.872 [20.25]	<0.0001	0.002

SDMA, symmetric dimethylarginine; SD, standard deviation; FDR, false discovery ratio.

**Table 3:** Linear model with covariate adjustment (limma) of significant AH metabolites.

	*p*-Value	Adjusted *p*-Value	B
SDMA	<0.0001	<0.0001	6.8453
Taurine	<0.0001	0.002	0.85187

SDMA, symmetric dimethylarginine; B, coefficient of interest.

**Table 4:** Correlation of axial length and AH metabolites significantly differentiating myopia and control groups.

	Correlation	t-Stat	*p*-Value	FDR
SDMA	−0.4401	193,040.0	<0.0001	<0.0001
Taurine	−0.4212	190,500.0	<0.0001	<0.0001

SDMA, symmetric dimethylarginine; t-stat, *t*-test for the significance of correlation; FDR, false discovery ratio.

**Table 5:** Correlation of taurine concentrations between AH and serum.

	Correlation	*p*-Value
Myopia	−0.1796	0.202662
Control	0.008671	0.95709

## Data Availability

The datasets from this research can be requested from the corresponding author.

## Supplementary Materials

The following supporting information can be downloaded at: https://www.mdpi.com/article/10.3390/ijms26178557/s1.
